# Twenty-seventh Annual Report of the Bloomingdale Asylum for the Insane

**Published:** 1848-02

**Authors:** 


ARTICLE X.

*Twenty-seventh Annual Report of the Bloomingdale Asylum for the Insane.* By PLINY EARLE, M. D., Physician to the Asylum, 1848.

*Fifth Annual Report of the Managers of the (New York) State Lunatic Asylum, made to the Legislature, Jan.* 1848.

*Report of the Pennsylvania Hospital for the Insane for the year* 1847. By THOMAS S. KIRKBRIDE, M. D., Physician to the Institution. Published by order of the Board of Managers, 1848.

The reports before us furnish most gratifying evidence of the prosperity of these institutions and show most conclusively, that by every years experience, new facts are developed with regard to the best mode of treating and managing this unfortunate class of our fellow beings.

Out of 274 cases there have been discharged from the Bloomingdale Asylum 58 cured, 17 much improved, 23 improved, 18 unimproved—total 116. New York State Asylum reports as discharged 1,137, in conditions as follows: recovered, 640; improved, 269; unimproved 114; died, 114. Total discharged from the Pennsylvania Hospital 213, as follows: cured, 111; much improved, 21; improved, 29; stationary 23; died, 29.

